# Wide Host Ranges of Herbivorous Beetles? Insights from DNA Bar Coding

**DOI:** 10.1371/journal.pone.0074426

**Published:** 2013-09-20

**Authors:** Keiko Kishimoto-Yamada, Koichi Kamiya, Paulus Meleng, Bibian Diway, Het Kaliang, Lucy Chong, Takao Itioka, Shoko Sakai, Motomi Ito

**Affiliations:** 1 Graduate School of Arts and Sciences, the University of Tokyo, Tokyo, Japan; 2 Faculty of Agriculture, Ehime University, Ehime, Japan; 3 Research, Development & Innovation Division, Sarawak Forest Department, Kuching, Sarawak, Malaysia; 4 Applied Forest Science and Industrial Development, Sarawak Forestry Corporation, Kuching, Sarawak, Malaysia; 5 Graduate School of Human and Environmental Studies, Kyoto University, Kyoto, Japan; 6 Research Institute for Humanity and Nature, Kyoto, Japan; University of California, Berkeley, United States of America

## Abstract

There are very few studies that have investigated host-specificity among tropical herbivorous insects. Indeed, most of the trophic interactions of herbivorous insects in Southeast Asian tropical rainforests remain unknown, and whether polyphagous feeding is common in the herbivores of this ecosystem has not been determined. The present study employed DNA bar coding to reveal the trophic associations of adult leaf-chewing chrysomelid beetles in a Bornean rainforest. Plant material ingested by the adults was retrieved from the bodies of the insects, and a portion of the chloroplast *rbcL* sequence was then amplified from this material. The plants were identified at the family level using an existing reference database of chloroplast DNA. Our DNA-based diet analysis of eleven chrysomelid species successfully identified their host plant families and indicated that five beetle species fed on more than two families within the angiosperms, and four species fed on several families of gymnosperms and/or ferns together with multiple angiosperm families. These findings suggest that generalist chrysomelid beetles associated with ecologically and taxonomically distant plants constitute a part of the plant-insect network of the Bornean rainforest.

## Introduction

An understanding of the host-specific interactions of herbivorous insects is critical to explaining the overwhelming diversity of plants and insects observed in tropical forests. For example, the finely partitioned niches promoted by the high host specificity of tropical insect herbivores could facilitate the species coexistence of these species (*see review* [1,2]). The concept of high host specificity among insects has also contributed to the estimation of total arthropod species richness on the earth since Erwin [3] first estimated the number of tropical arthropod species at 30 million [4,5]. However, recent empirical studies have demonstrated that host specificity among herbivorous insects to particular plant species may be less common than previously believed. In particular, polyphagous feeding on congeneric and confamilial plant species is common among leaf chewers (e.g., [5–11]). However, these results come primarily from Neotropical and Papua New Guinean forests, and it remains unclear whether polyphagous feeding is common for herbivorous insects in other tropical regions.

Rainforests in the central part of Southeast (SE) Asia are characterized by the most humid and aseasonal climate of the ecosystems in the region [12]. In the lowlands of the region, the Dipterocarpaceae are the dominant plant group in terms of both species richness and abundance [13,14]. Tropical forests tend to be dominated by highly species-rich genera and families of trees [15], and in such forests, most herbivores feed on multiple plant species within these genera and families (e.g., [8,15,16]). Therefore, it is likely that polyphagous feeding within the Dipterocarpaceae prevails among herbivorous insects in the region. However, due to the low availability of preferable food resources obtainable from dipterocarp trees, Dipterocarpaceae specialists may be uncommon among these herbivores. Previous studies have demonstrated that many herbivores prefer young leaves and flowers over mature leaves in the tropical forest canopy (e.g., [17–20]). The leaf and flower production of many dipterocarp trees is known to fluctuate synchronously among species at irregular intervals [13, 14, 21–23]. In contrast, the adults of some leaf chewer species emerge constantly throughout the year and fluctuate in abundance independently of dipterocarp tree phenology [23,24], although they have been observed to feed on the young leaves and flowers of dipterocarp trees [25]. This pattern leads us to hypothesize that these leaf chewer species are able to feed on other plant species when the young leaves and flowers of dipterocarp trees are unavailable. However, previous studies have focused primarily on distribution of herbivores on locally common plants, including dipterocarp trees [25,26], and the ability of some herbivores to feed not only on common plants but also on other species had not been confirmed.

Sampling a variety of plants from the local flora is a common methodology to study the trophic interactions of herbivores and infer host-specialization or generalization. Investigations involving the host specializations of tropical herbivores have often relied on the direct observation of insect feeding, the experimental verification of this behavior, and insect rearing [1]. These methods necessarily restrict the target plants from which herbivorous insects are captured. Indeed, previous studies have sampled only a portion of the local flora [1,2], with species often chosen based on plant phylogeny [16,27,28], ecological importance [7], or local abundance [6,8,25,26,29]. Alternatively, DNA bar coding, the taxonomic identification of species using short DNA sequence markers, has received considerable attention in the past several years as a method for determining the dietary compositions of organisms [30–33]. These studies have extracted DNA from the plant tissues ingested by insect herbivores by processing part or all of bodies of the insects [30–33]. A portion of the chloroplast DNA was then amplified, and the plant sequences were identified through comparison to an existing reference database such as GenBank [30,31] or the reference DNA bar code library of the target plant groups [33]. This DNA-based technique enables us to determine the host plants of insect herbivores without direct feeding observations. Moreover, because the technique is less targeted, it reduces the possibility that the trophic relationships of generalist herbivores will be overlooked. However, few studies have applied this DNA-based technique to reveal the host plants of those herbivorous insects for which the host plants are completely unknown.

The adults of Chrysomelidae, known to be leaf chewers, are one of the dominant groups foraging in tropical forest canopies [34–36]. The host plants of only approximately 20% of the 37,000 described chrysomelids are known [37], and most of their trophic relationships are unknown in the tropics. This study aimed to identify the plant families that chrysomelid adults (Galerucinae, Chrysomelidae) feed on in a Bornean rainforest and to evaluate the use of DNA bar coding to understand the interactions between herbivorous insects and their unknown host plants in a Bornean tropical forest.

## Materials and Methods

### Study area

The field survey was conducted in the Canopy Biology Plot (CBP, 200×400 m; [38]) at Lambir Hills National Park (LHNP), Sarawak, Malaysia (4^°^20´ N, 113^°^50´ E; 150–200 m a.s.l.). The park covers an area of approx. 6,949 hectares [38], and also included the 52-ha plot (1,040×500 m; approx. 2.5 km at southwest of the CBP) and the Crane Plot (200×200 m; approx. 500 m at northeast of the CBP [38]). The family Dipterocarpaceae dominates the canopy and emergent layers in the CBP, whereas Euphorbiaceae, Burseraceae, and Myristicaceae are dominant in the lower vegetation layers [39]. The mean annual temperature is approx. 27°C [40]. The mean annual rainfall in the park for the period of 2000–2006 was approx. 2600 mm [40], and there was no defined dry season [39].

At the CBP, community-level plant phenology has been monitored for 576 individual plants, comprising 305 species in 56 families [41]. From 1994–1997, the proportion of flushing trees, defined as trees with more than 10% of all crown leaves in the newly developing or immature stages, ranged from 3.8 to 30.1% [23], and flushing trees increased irregularly as 14- and 30-day cumulative rainfall decreased [22,23]. In addition, a number of community-wide synchronous flowering periods, defined as periods during which more than 6% of individual canopy trees undergo flowering, occurred irregularly at intervals of several years, and the number of flowering trees approached zero during non-flowering periods [21,42].

This study was conducted in accordance with the Memorandum of Understanding between the Sarawak Forestry Corporation and the Japan Research Consortium for Tropical Forests in Sarawak signed in November 2005.

### Insect Sampling

Chrysomelid adults were collected using light traps. Chrysomelidae were the most abundant group of light-trapped beetles at the study site [34]. The adults were manually collected from 18:30 to 22: 00 using a 20W blacklight tube (FL20SBLB, National, Japan) on the towers and aerial walkways (15–35 m above the ground) constructed in the CBP. The collection was conducted on several days near the new moon of September 2009, February and April 2010, and February, August, and November 2011. Each chrysomelid adult was placed in a separate screw cap tube filled with 99.5% ethanol immediately after being captured. A preliminary test indicated that all 20 adults of *Gastrophysa* *atrocyanea* (Chrysomelinae, Chrysomelidae) that had been reared with their host plants regurgitated when they were placed in 99.5% ethanol, whereas all 20 adults that had been reared without any food materials for a few days did not regurgitate when placed in ethanol. Therefore, only those individuals that regurgitated material when they were placed in ethanol were used for DNA extraction.

### DNA data collection

DNA was extracted from the head and thorax of each of the 72 light-attracted chrysomelid adults using a DNeasy Blood & Tissue Kit (Qiagen, Maryland, USA) [30]. The beetle specimens used for DNA extraction were mounted as voucher specimens and are maintained at Forest Research Centre. The chrysomelids were discriminated into morphospecies and identified on the basis of external characteristics, with reference to a collection of specimens used for previous studies [23,24].

The DNA template extracted from each individual was used for two purposes: 1) to discriminate the 72 chrysomelid specimens into species in combination with our morphological examination and 2) to identify the plants that each chrysomelid adult had fed on. For discriminating the chrysomelid species, the universal primer pair LCO1490 (5′-GGTCAACAAATCATAAAGATATTGG-3′) and HCO2198 (5′-TAAACTTCAGGGTGACCAAAAAATCA-3′) was used to amplify a 658-bp fragment of the mitochondrial *COI* gene [43]. The final reaction solution (20 µl) of each PCR amplification consisted of 2 µl 10× Ex Taq Buffer, 1.6 µl 2.5 mM dNTP mixture, 0.5 units ExTaq DNA polymerase (Takara Bio, Otsu, Japan), 0.5 µl each primer (20 µM), 14.3 µl autoclaved distilled water, and 1 µl DNA template. The PCR thermal program consisted of 1 min at 94°C, 35 cycles of 30 sec at 94°C, 30 sec at 50°C, and 1 min at 72°C, and a final extension of 10 min at 72°C. The PCR products were sequenced in both directions using an Applied Biosystems 3730*xl* DNA analyzer. The sequence chromatograms were visually inspected, and the reads in the forward and reverse directions were assembled using ATGC version 6.04 (Genetyx, Tokyo, Japan). A 650–658-bp of sequence was determined for each individual, and the multiple alignment sequences dataset for subsequent analyses involved 644 bp. All of the sequences were deposited in the DNA Data Bank of Japan (DDBJ) under the accession numbers listed in Table S1.

In addition, the same DNA samples were used to amplify plant plastid DNA. The partial region of chloroplast *rbcL* was amplified using PCR primers, namely *rbcLa* forward (5′-ATGTCACCACAAACAGAGACTAAAGC-3′) and *rbcLa* reverse (5′-GTAAAATCAAGTCCACCRCG-3′) [44]. The PCR solution (20 µl) for each PCR amplification consisted of 2 µl 10× Ex Taq Buffer, 1.6 µl 2.5 mM dNTP mixture, 0.5 units ExTaq DNA polymerase (Takara Bio, Otsu, Japan), 0.5 µl each primer (10 µM), 13.3–14.3 µl autoclaved distilled water and 1–2 µl DNA template. We used the following standard PCR temperature profile for bar coding [44]: 4 min at 94°C; 35–40 cycles of 30 sec at 94°C, 1 min at 55°C, 1 min at 72°C; 10 min at 72°C; and a hold at 10°C. Because it was possible for some chrysomelid individuals to have ingested more than one plant species, we conducted a single-strand conformation polymorphism (SSCP) analysis to detect the PCR amplicons of multiple plant species in a single chrysomelid individual. This method can detect small DNA changes, such as single base substitutions, deletions, and insertions [45,46]. Two bands were detected on the SSCP gel when a given chrysomelid individual had ingested only one plant species, whereas four bands were detected on the gel when an individual had ingested two plant species. We used a portion of the PCR products for the SSCP gels [46,47], the composition of which was based on 0.5×MDE gel solution (Lonza, Rockland, USA) and included 2, 5, or 7% glycerol. Electrophoresis was performed in 0.5×TBE (45 mM Tris-borate and 1 mM EDTA·2Na) at 300 V for 12 hr using an electrophoretic apparatus with a thermostat-controlled cooled water circulator (AE-6290, ATTO, Tokyo, Japan); the gel temperature was maintained at 20°C. After electrophoresis, the SSCP bands were detected using the following silver staining protocols: 1) fixation -10% acetic acid (30 min); 2) wash - water (2 min, 3 times); 3) staining -1% AgNO_3_, formalin, water (20 min); 4) wash - water (30 sec); 5) development - Na _2_CO_3_, 2% Na _2_S_2_O_3_·5H_2_O, formalin, water (until dark-staining bands appeared on the yellow background of the SSCP gels); and 6) stop - EDTA·2Na, water (10 min). One band of a pair was retrieved from the dried SSCP gels and PCR amplified a second time using the same procedure. The second PCR products were sequenced in both directions using an Applied Biosystems 3730*xl* DNA analyzer. The sequence chromatograms were assembled and edited with ATGC version 6.04 (Genetyx, Tokyo, Japan), resulting in a 547–553-bp sequence per individual. The sequences in the dataset were trimmed to 547 bp for the subsequent analyses. All of the sequences were deposited in DDBJ (Table S1).

### Discrimination of chrysomelid and plant species

To discriminate the *COI* sequences into species, a neighbor-joining (NJ) tree was constructed based on Kimura’s 2-parameter (K2P) genetic distance [48] using MEGA5 [49]. Because the *rbcLa* sequence showed insufficient variation to distinguish among closely related species [44,50], any unique *rbcLa* sequence was treated as an operational taxonomic unit (OTU). An NJ tree was also constructed using *rbcLa* sequences to show the phylogenetic relationships among the plant families.

### Plant family estimation

To estimate which plant family each chrysomelid adult had fed on, 1) each *rbcLa* sequence was compared against the GenBank nr database, and 2) the plant family with the most similar sequence was then compared with the plant lists for the LHNP. In the first procedure, we used Claident ver. 0.1.2012.09.03, which is a software package designed for rapid sequence classification and the accurate taxonomic identification of host organisms from sequences for bar coding studies (http://www.fifthdimension.jp/products/claident/). This identification system is based on the idea that the similarity between a query sequence and the nearest-neighbor sequence must be higher than the minimum similarity value within the taxonomic group to which the nearest-neighbor sequence belongs (A.S. Tanabe, unpublished).

In the second procedure, we used the plant lists available for 1) trees with >1 cm dbh (diameter breast height) in the 52-ha plot [51], 2) trees with >5 cm dbh in CBP, 3) trees with >5 cm dbh in the Crane Plot, and 4) vascular plants (dicotyledons, monocotyledons, gymnosperms, ferns, and fern allies) collected primarily from the CBP [52].

## Results

### Species discrimination of chrysomelids

Seventy-two *COI* sequences were discriminated into eleven chrysomelid species (Table 1). The intraspecific distances for each chrysomelid species corroborated the species discriminations based on a morphological basis: the intraspecific distances were less than 2% for all species (Table 1). This result is compatible with the tendency of intraspecific distances based on K2P distances to be less than 2% for the *COI* bar coding region of beetles [53–57].

**Table 1:** Summary of the intraspecific Kimura 2-parameter distances for chrysomelid species.

Species	Species code	No of adults	Extracted plant sequences	Intra K2P distance range
*Anadimonia* sp ?	An *	6	7	0.000
*Hyphaenia* sp.	Hy3	7	10	0.005-0.016
*Liroetiella* *antennata*	Li1 *	7	7	0.000-0.014
*Monolepta* sp. 1	Mo2 *	2	2	0.000
*Monolepta* sp. 2	Mo3 *	3	4	0.005-0.011
*Monolepta* sp. 3	Mo4 *	9	9	0.000-0.014
*Monolepta* sp. 4	Mo5 *	11	14	0.000-0.006
*Monolepta* sp. 5	Mo7 *	2	2	0.000
*Monolepta* sp. 6	Mo16	4	4	0.000-0.005
*Monolepta* sp. 7	Mo17	9	12	0.000-0.019
*Theopea* sp.	Th *	12	15	0.000-0.009

Species codes of species with asterisks (*) corresponded with those in previous studies [23,24].

### Inferred Plant Family

**Figure 1:**
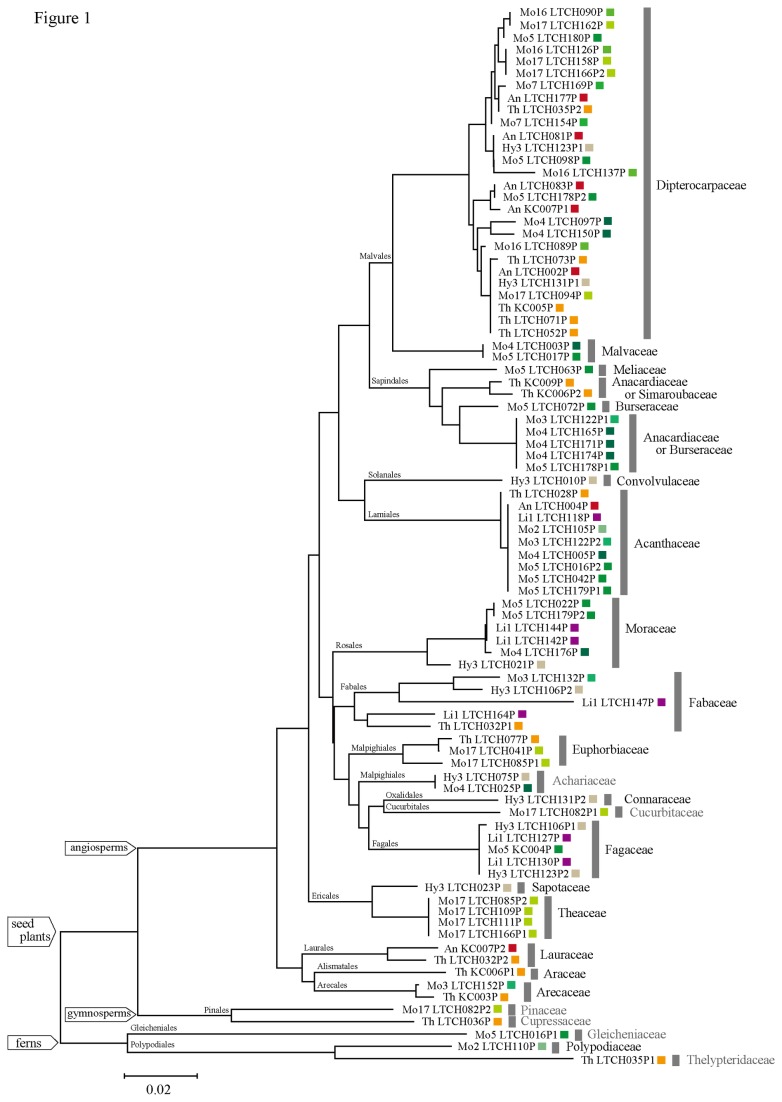
Neighbor-joining tree based on the *rbcL*a region sequences retrieved from chrysomelid bodies. A gray bar indicates a plant family. The family name in black indicates that the family is known to occur at the study site, while the name in gray indicates the family was not recorded at the study site. The sequence codes are listed in Table S1. Each color indicates a different chrysomelid species, and the species codes are listed in Table 1.

Eighty-six plant sequences detected among the 72 chrysomelid individuals were discriminated into 53 OTUs. The 53 OTUs were assigned to at least 24 families in 18 orders (Figure 1); of these 53 OTUs, 48 were assigned to 19 angiosperm families. Of these, 15 OTUs were determined to belong to Dipterocarpaceae. Five OTUs were determined to belong to the order Sapindales; one OTU each belonged to Meliaceae and Burseraceae, and the others did not clearly match a single plant family (Anacardiaceae/Simaroubaceae or Anacardiaceae/Burseraceae). Five OTUs were determined to belong to Fabaceae; four to Moraceae; three to Euphorbiaceae; two each to Lauraceae, Arecaceae, Fagaceae, and Acanthaceae; and one OTU each to Araceae, Connaraceae, Malvaceae, Sapotaceae, Theaceae, and Convolvulaceae. All of these families occurred at the study site. Although one OTU was determined to belong to Cucurbitaceae and one to Achariaceae, these families have not been recorded at the study site. In addition, two OTUs were determined to belong to gymnosperm families, Pinaceae and Cupressaceae, and these families do not occur at the study site. The Claident search result indicated that one sequence (Mo17 LTCH082P2; Figure 1) belonged to the genus *Pinus* (Pinaceae); the sequence (Th_LTCH036P; Figure 1) of the other species was identical to the chloroplast sequence of *Chamaecyparis* *obtusa* . Moreover, one sequence was assigned to each of three families of ferns (Gleicheniaceae, Thelypteridaceae, and Polypodiaceae), even though only Polypodiaceae has been recorded to occur at the study site.

### Host plant ranges of chrysomelid individuals and species

**Figure 2:**
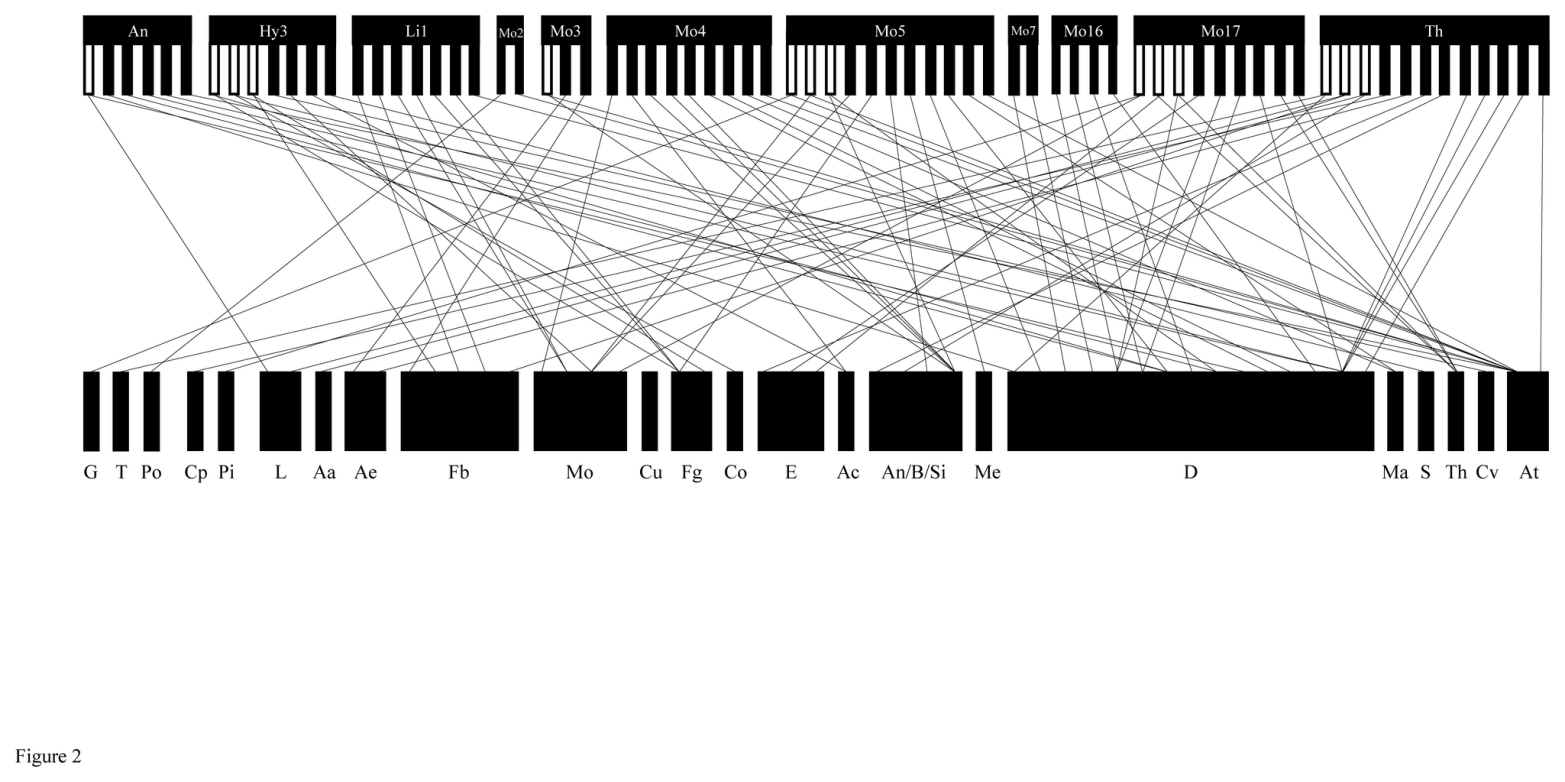
Plant–herbivore interactions in the study site. The lower bars indicate the following plant families: G, Gleicheniaceae; T, Thelypteridaceae; P, Polypodiaceae; Cp, Cupressaceae; Pi, Pinaceae; L, Lauraceae; Aa, Araceae; Ae, Arecaceae; Fb, Fabaceae; Mo, Moraceae; Cu, Cucurbitaceae; Fg, Fagaceae; Co, Connaraceae; E, Euphorbiaceae; Ac, Achariaceae; An, Anacardiaceae; B, Burseraceae; Si, Simaroubaceae; Me, Meliaceae; D, Dipterocarpaceae; Ma, Malvaceae; S, Sapotaceae; Th, Theaceae; Cv, Convolvulaceae; and At, Acanthaceae. The upper bars indicate the individual of eleven chrysomelid species. The species codes are listed in Table 1. For the upper bars, a white bar indicates the individual ingested two plant species, and a black bar indicates that the individual ingested one plant species.

Our PCR-SSCP analysis, followed by DNA sequencing, determined that two plant sequences were obtained from a single individual for 14 chrysomelid individuals. For each of these 14 individuals, the adult fed on two plant species belonging to different families (Table 2, Figure 2).

**Table 2:** The inferred host plants of 11 chrysomelid species.

Species code	No of sequences	No of species	Inferred plant family
An	1	1	Lauraceae
	5	5	Dipterocarpaceae
	1	1	Acanthaceae
Hy3	1	1	Moraceae
	2	2	Fagaceae
	1	1	Connaraceae
	1	1	Achariaceae
	2	2	Dipterocarpaceae
	1	1	Sapotaceae
	1	1	Convolvulaceae
Li1	2	1	Fagaceae
	2	1	Moraceae
	2	2	Fabaceae
	1	1	Acanthaceae
Mo2	1	1	Acanthaceae
	1	1	Polypodiaceae
Mo3	1	1	Arecaceae
	1	1	Fabaceae
	1	1	Anacardiaceae / Burseraceae
	1	1	Acanthaceae
Mo4	1	1	Moraceae
	1	1	Achariaceae
	2	2	Dipterocarpaceae
	1	1	Malvaceae
	3	1	Anacardiaceae / Burseraceae
	1	1	Acanthaceae
Mo5	1	1	Fagaceae
	2	1	Moraceae
	3	3	Dipterocarpaceae
	1	1	Malvaceae
	1	1	Burseraceae
	1	1	Anacardiaceae / Burseraceae
	1	1	Meliaceae
	3	1	Acanthaceae
	1	1	Gleicheniaceae
Mo7	2	2	Dipterocarpaceae
Mo16	4	4	Dipterocarpaceae
Mo17	1	1	Cucurbitaceae
	2	2	Euphorbiaceae
	4	3	Dipterocarpaceae
	4	1	Theaceae
	1	1	Pinaceae
Th	1	1	Lauraceae
	1	1	Arecaceae
	1	1	Araceae
	1	1	Fabaceae
	1	1	Euphorbiaceae
	5	3	Dipterocarpaceae
	2	2	Anacardiaceae / Simaroubaceae
	1	1	Acanthaceae
	1	1	Cupressaceae
	1	1	Thelypteridaceae

The total number of inferred host plant families per species ranged from one to ten (Table 2). For three *Monolepta* species (Mo17, Mo5, and Mo2) and one *Theopea* species (Th), the adults fed on plant species of multiple subdivisions or even divisions (Figures 1 and 2). Two chrysomelid species were associated with only Dipterocarpaceae host plants.

## Discussion

Our dietary analysis of eleven chrysomelid species using DNA bar coding indicates that the host ranges of nine of these species were remarkably wide. Five beetle species fed on more than two families within the angiosperms, and four species fed on several families of gymnosperms and/or ferns together with multiple angiosperm families (Table 2). Previous studies have demonstrated that several species of leaf chewers feed on different families of angiosperm plants [6–8]. However, feeding on multiple plant families across subdivisions or divisions appears to be rare. Even monocots and other angiosperm families do not share the majority of herbivore communities [28]. Our results therefore indicate a surprisingly generalist behavior among several species of leaf-chewing chrysomelids in a Bornean rainforest. These findings contrast with earlier estimates of host specificity among tropical insect herbivores [1,2,3] and even more recent studies of herbivore host range over plant phylogeny [16,27,28]. However, our study explored only a portion of the overwhelming diversity of plant-herbivorous insect interactions present in the SE Asian tropics. Whether such generalist behavior is also common for other herbivorous insects in this ecosystem remains to be seen.

Our findings are not consistent with the hypothesis that highly specialized herbivores maintain the diversity of tropical trees. Density-dependent effects are hypothesized to be caused by specialized herbivores or pathogens that damage the conspecific trees in the Janzen-Connell hypothesis (*see review* [58]). To date, there exist a few studies which have found that these density-dependent processes contribute to the coexistence of tree species in a Bornean rainforest [59,60]. However, our study implies that the density-dependent effects are unlikely to be frequently caused by leaf-chewing chrysomelids in our forest, because generalist herbivores do not have a tendency to concentrate their host plant use on a particular plant species. Thus, if natural enemies are causing density-dependent patterns in this forest, they are likely to be soil pathogens or other insect herbivores besides the eleven beetle species in our study.

The present study successfully identified the host plant families for eleven chrysomelid species, although six of the identified plant families were not recorded at the study site. Because several genera of Cucurbitaceae, Gleicheniaceae, and Thelypteridaceae are commonly found on the island of Borneo [61,62], it is likely that species of these families occur in and around the study site and that chrysomelid adults feed on these families. In addition, 30 genera previously belonging to the Flacourtiaceae have been incorporated into the revised Achariaceae [63]. Of these genera, the genus *Ryparosa* (recorded as Flacourtiaceae) is common at the study site, suggesting that several chrysomelid individuals fed on *Ryparosa* or allied species. Moreover, our finding that the sequence extracted from one *Theopea* adult was identical to the chloroplast sequence of *Chamaecyparis* *obtusa* suggests that adult beetles feed on *C* *. obtusa* , which is a commonly planted species in Sarawak [64]. Several conifer species, such as *Pinus*, are also planted widely in Sarawak [64,65]. Because chrysomelid adults are known to be good fliers [66] and the CBP, where we collected the species, was located near the edge of the primary forest [38], the beetles likely fed on trees that were planted in neighboring lands outside the primary forest research plot.

It is likely that the Dipterocarpaceae are the main host plant family for the chrysomelid adults (Figures 1 and 2), as this group is dominant at the study site in terms of both species richness and abundance [39]. Our data also supported the hypothesis that Dipterocarpaceae specialists are not common among the studied insect species. For example, nine beetle species fed on several plant families including canopy tree species, such as Anacardiaceae, Burseraceae, Euphorbiaceae, Fabaceae, Fagaceae, Lauraceae, Meliaceae, Moraceae, Sapotaceae, and Simaroubaceae [41]. At the study site, several species of Dipterocarpaceae and other canopy trees synchronously flushed their leaves [22]. The chrysomelid adults may feed on the young leaves of other canopy species along with those of dipterocarp trees during these synchronous flushing events. In addition to canopy trees, our findings suggest that the inferred host plants of the chrysomelid adults include a number of growth forms, such as herbs, shrubs, and vines. Surprisingly, several adults also fed on ferns. Taken together, these findings indicate that these chrysomelid adults may broadly select food resources from the local flora to sustain their populations throughout the year. However, our sample size was limited, and the identification of host plants using DNA bar coding is limited to at the family level. The effects of interspecific variations in plant ecological traits such as phenology, defense against herbivory, and growth form on the host plant choices of these chrysomelid adults should be investigated in further detail.

This study proposes an effective approach for studying the trophic associations of generalist herbivores. Previous studies on the host plant-herbivorous insect interactions of tropical forests have targeted only a portion of the local flora [1,2]. Though they are renowned for their species-richness, tropical forests are often characterized by a small number of species-rich genera and families that contribute disproportionately to local diversity [67]. Most studies have focused mainly on these locally common plants [6,8,25,26,29]. Our study indicates that several chrysomelid adults consumed not only locally dominant species but also other plant families. This finding suggests that a DNA-based method may enhance the detection of the host plants of generalist herbivores. In addition, the inferred host plant families of the chrysomelids included various growth forms, such as canopy trees, herbs, shrubs, and vines, indicating that chrysomelid adults are able to forage both in the canopy and the forest floor. This finding is incompatible with the previously observed tendency of herbivorous insects to use a particular vertical stratum of their habitat preferentially, as suggested by the low similarity of herbivorous fauna recorded between the canopy and the floor in tropical forests ( [34,35] *but* see [36]). If the target flora in previous studies were selected based on the presumed foraging habitats of herbivores, the host ranges of these species may be underestimated. Therefore, mass collections using traps and subsequent DNA-based diet analysis may place fewer constraints on the target plants and habitats of future studies.

Studies using plant DNA sequences extracted from insect guts are effective when these sequences can be compared with a plant DNA bar code reference library for the host plants of the focal study area [33]. However, such reference libraries, especially the databases covering local flora, are still incomplete in many tropical forests. The Consortium for the Barcode of Life (CBOL) working group has proposed the use of a 2-locus combination of *rbcL* + *matK* for bar coding due to its universality, sequence quality, and species discrimination [68]. For tropical plants, the cpDNA representation of these loci in databases is expected to increase rapidly, leading to improved accuracy and speed in host plant identification [44,69,70]. Considering the expected improvements in the representation of the cpDNA loci proposed by the CBOL working group in databases for tropical areas, the locus used in the present study is likely to be useful for future investigations of plant-insect food webs in tropical forests. A recent study also demonstrated that the *rbcL* sequences retrieved from the gut contents of rolled-leaf beetles successfully identified the families of their host plants [33]. For identification at the genus or species level, a combination of short genic regions of cpDNA and the construction of a comprehensive DNA bar code library for the study site will be required [33].

A cloning step must be performed to identify multiple host plants from the gut of one insect individual, as polyphagous herbivores move frequently between host plants [33]. To solve this problem, we performed PCR-SSCP analysis. This method effectively determined 1–2 plant species per chrysomelid individual, and each PCR product excised from the SSCP gels was successfully re-amplified and used for DNA sequencing. The SSCP-based procedure used in this study for discriminating multiple plant species ingested by a single chrysomelid individual may be easier and more cost-effective than the cloning method [30], which often incorporates PCR errors. Alternatively, DNA metabarcoding may provide a solution to this problem in the near future [71].

The determination of the appropriate mass collection method for DNA-based diet analysis is important to obtain useful data A previous study of unknown plant–herbivore interactions argued that the amplification of plant DNA from trap-caught insects frequently fails, because the plant DNA in the insect gut degrades over several days in the preservative of the trap [31]. To prevent this problem, each of our specimens was placed in a separate screw cap tube with ethanol immediately after capture, which may have enhanced the detectability of the plant DNA ingested by the chrysomelid adults. The collection of chrysomelid adults with lights was useful in this study because the beetles were observed both during the day and at night [25], and the Chrysomelidae were the most abundant group among the light-trapped beetles at the study site [34]. However, this method is generally not useful for the collection of diurnal flying insects, which includes a large number of herbivores. Mass collection methods must be selected for their ability to target specific herbivore guilds or species and to lessen the damage to the plant DNA ingested by the insects.

In conclusion, this study proposes an effective approach for studying the trophic associations of insect herbivores at the plant family level in tropical forests. Our findings suggest that generalist herbivores associated with ecologically and taxonomically distant plants constitute a part of the plant-insect network of the Bornean rainforest. These observations contrast with recent studies of herbivore host ranges over plant phylogeny [16,27,28] and with older estimates of extremely narrow host ranges among tropical herbivores [3]. Previous studies may have underestimated the host ranges of insect herbivores and overlooked the host generality of insect herbivores in tropical forests, and our technique may reduce these possibilities because it is less targeted. Alternatively, this study is based on a very small sample size, and thus the results could be an anomaly. The construction of a more complete DNA bar code reference database for the plant species in the target study area, in combination with improvements in the methods of DNA-based analysis, will increase the accuracy of host plant identification, enabling us to construct a more comprehensive plant-insect network.

## Supporting Information

Table S1 **Voucher specimens of chrysomelids and the accession numbers of the *COI* region and the *rbcLa* region extracted from each chrysomelid.**
(PDF)
