# Supplementary material for: Wide Host Ranges of Herbivorous Beetles? Insights from DNA Bar Coding
Source: PLoS One. 2013 Sep 20;8(9):e74426. doi: 10.1371/journal.pone.0074426 (PMC3779210; doi:10.1371/journal.pone.0074426)
Supplement: Table S1 — Voucher specimens of chrysomelids and the accession numbers of the COI region and the rbcLa region extracted from each chrysomelid. (PDF) [file pone.0074426.s001.pdf]

Table S1. Voucher specimens of chrysomelids and the accession numbers of the *COI* region and the *rbcLa* region extracted from each chrysomelid.

Individual ID	*COI* accession number	*rbcLa* accession number
Anadimonia sp.? KKY2013 (An)		
KC007	AB794707	AB794779, AB794780
LTCH002	AB794708	AB794781
LTCH004	AB794709	AB794782
LTCH081	AB794710	AB794783
LTCH083	AB794711	AB794784
LTCH177	AB794712	AB794785
Hyphaenia sp. KKY2013 (Hy3)		
LTCH010	AB794713	AB794786
LTCH021	AB794714	AB794787
LTCH023	AB794715	AB794795
LTCH075	AB794716	AB794788
LTCH106	AB794717	AB794789, AB794790
LTCH123	AB794718	AB794791, AB794792
LTCH131	AB794719	AB794793, AB794794
Liroetiella antennata (Li1)		
LTCH118	AB794720	AB794796
LTCH127	AB794721	AB794797
LTCH130	AB794722	AB794798
LTCH142	AB794723	AB794799
LTCH144	AB794724	AB794800
LTCH147	AB794725	AB794801
LTCH164	AB794726	AB794802
Monolepta sp. KKY2013-1 (Mo2)		
LTCH105	AB794727	AB794819
LTCH110	AB794728	AB794820
Monolepta sp. KKY2013-2 (Mo3)		
LTCH122	AB794729	AB794821, AB794822
LTCH132	AB794730	AB794823
LTCH152	AB794731	AB794824
Monolepta sp. KKY2013-3 (Mo4)		
LTCH003	AB794732	AB794825
LTCH005	AB794733	AB794826
LTCH025	AB794734	AB794827
LTCH097	AB794735	AB794828
LTCH150	AB794736	AB794829
LTCH165	AB794737	AB794830
LTCH171	AB794738	AB794831
LTCH174	AB794739	AB794832
LTCH176	AB794740	AB794833
Monolepta sp. KKY2013-4 (Mo5)		
KC004	AB794741	AB794834
LTCH016	AB794742	AB794835, AB794836
LTCH017	AB794743	AB794837
LTCH022	AB794744	AB794838
LTCH042	AB794745	AB794839
LTCH063	AB794746	AB794840
LTCH072	AB794747	AB794841
LTCH098	AB794748	AB794842
LTCH178	AB794749	AB794843, AB794844

LTCH179	AB794750	AB794845, AB794846
LTCH180	AB794751	AB794847
Monolepta sp. KKY2013-5 (Mo7)		
LTCH154	AB794752	AB794848
LTCH169	AB794753	AB794849
Monolepta sp. KKY2013-6 (Mo.16)		
LTCH089	AB794754	AB794803
LTCH090	AB794755	AB794804
LTCH126	AB794756	AB794805
LTCH137	AB794757	AB794806
Monolepta sp. KKY2013-7 (Mo.17)		
LTCH041	AB794758	AB794807
LTCH082	AB794759	AB794808, AB794809
LTCH085	AB794760	AB794810, AB794811
LTCH094	AB794761	AB794812
LTCH109	AB794762	AB794813
LTCH111	AB794763	AB794814
LTCH158	AB794764	AB794815
LTCH162	AB794765	AB794816
LTCH166	AB794766	AB794817, AB794818
Theopea sp. (Th)		
KC003	AB794767	AB794850
KC005	AB794768	AB794851
KC006	AB794769	AB794852, AB794853
KC009	AB794770	AB794854
LTCH028	AB794771	AB794855
LTCH032	AB794772	AB794856, AB794857
LTCH035	AB794773	AB794858, AB794859
LTCH036	AB794774	AB794860
LTCH052	AB794775	AB794861
LTCH071	AB794776	AB794862
LTCH073	AB794777	AB794863
LTCH077	AB794778	AB794864
